# Data on the *Amphidinium carterae* Dn241EHU isolation and morphological and molecular characterization

**DOI:** 10.1016/j.dib.2018.07.036

**Published:** 2018-07-21

**Authors:** S. Seoane, A. Molina-Miras, L. López-Rosales, A. Sánchez-Mirón, M.C. Cerón-García, F. García-Camacho, I. Madariaga, E. Molina-Grima

**Affiliations:** a Plant Biology and Ecology Department, University of the Basque Country (UPV/EHU), 48940 Leioa, Spain; b Technology and Research Centre for Experimental Marine Biology and Biotechnology (PiE-UPV/EHU), 48620 Plentzia, Spain; c Chemical Engineering Area, University of Almería, 04120 Almería, Spain; d Research Center in Agrifood Biotechnology, University of Almería, 04120 Almería, Spain

## Abstract

We present the data corresponding to the isolation and morphological and molecular characterization of a strain of *Amphidinium carterae*, isolated in Mallorca Island waters and now deposited in the microalgae culture collection of the Plant Biology and Ecology Department of the University of the Basque Country under the reference Dn241Ehu. The morphological characterization was made using two different techniques of microscopy and the molecular characterization by using the 28S rDNA sequences of D1 and D2 domains. This strain has been used for a culture study in an indoor LED-lighted pilot-scale raceway to determine its production of carotenoids and fatty acids, “Long-term culture of the marine dinoflagellate microalga *Amphidinium carterae* in an indoor LED-lighted raceway photobioreactor: Production of carotenoids and fatty acids.” (Molina-Miras et al., 2018) [1].

**Specifications Table**

**Table:** 

Subject area	*Biotechnology of Microalgae*
More specific subject area	*Phycology*
Type of data	*Morphological feature images and molecular analysis*
How data was acquired	*Morphological features: Light and confocal microscopy*
	*Molecular analysis: Sanger dideoxy sequencing-ABI PRISM 3130xl Genetic Analyzer. Phylogenetic tree development explained in the text of this article*
Data format	*Morphological features: images*
	*Molecular analysis: Raw sequence reads and phylogenetic tree*
Experimental factors	*Culture conditions (explained in the text of this article)*
Experimental features	*Morphological characterization was made using two different techniques of microscopy and the molecular characterization by using the 28S rDNA sequences of D1 and D2 domains.*
Data source location	*The strain Dn241EHU was isolated from Punta des Gas, Mallorca,* Spain (39°33׳50"N - 2°39׳20"E)
Data accessibility	*Data incorporated within this article and the sequence of Amphidinium carterae Dn241EHU has been deposited in GenBank under the accession number*MG520273
Related research article	*Long-term culture of the marine dinoflagellate microalga Amphidinium carterae in an indoor LED-lighted raceway photobioreactor: Production of carotenoids and fatty acids*. Bioresource Technology, **265**: 257–267.

**Value of the data**

• The data provide an example of isolation and conditions of cultivation of *Amphidium* species.
• Important morphological features for the identification of *Amphidinium carterae* are described.
• 28S rDNA D1-D2 domains are sequenced and the data provide the position of this strain in an *Amphidinium* genus phylogenetic tree.

## 1 Data

**Fig. 1:**
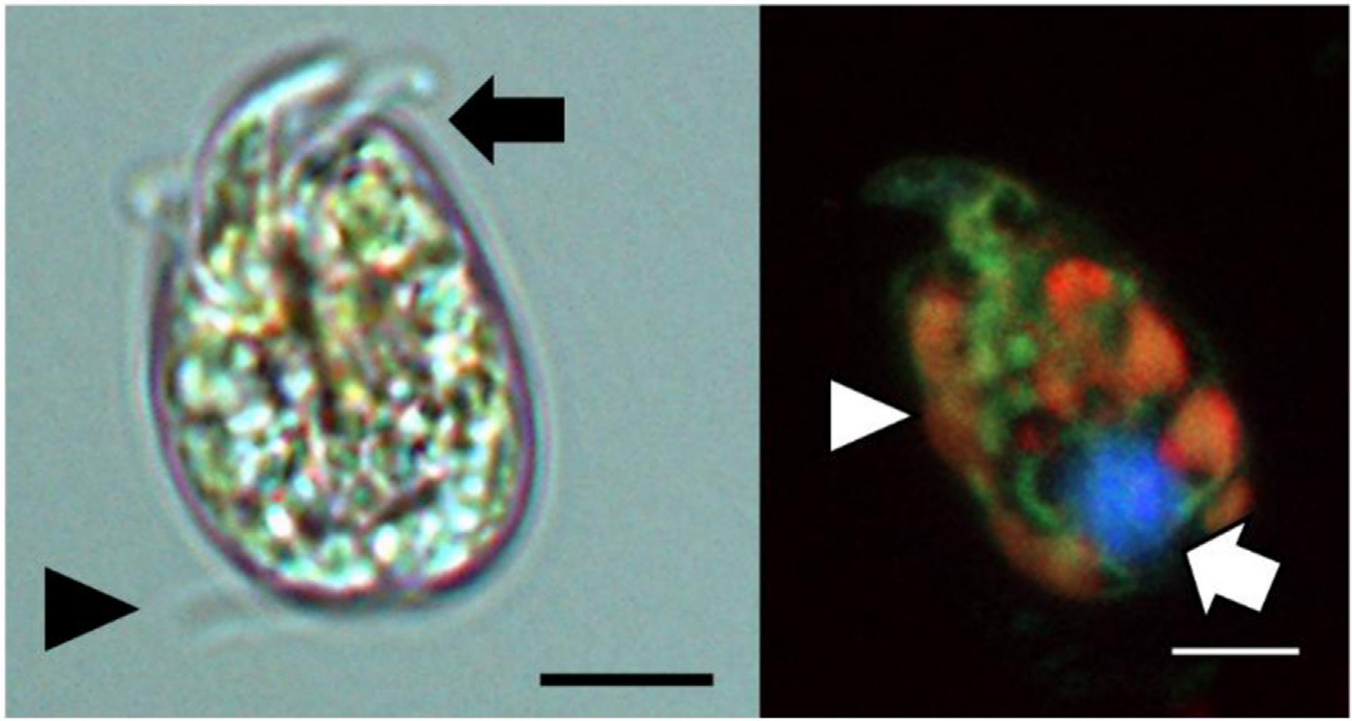
Micrographs of *Amphidinium carterae* Dn241EHU from Punta des gas (Palma de Mallorca, Spain). A) Light microscopy, showing the anterior flagellum (arrow) and the posterior flagellum (arrow head); B) Confocal microscopy, showing the nucleus (arrow) and multi-lobed chloroplast (arrow head). Scale bar: 5 µm.

The morphology of the *Amphidinum carterae* Dn241EHU strain shows the typical shape of *Amphidinium* genus with a small epicone and big hypocone (Fig. 1). The epicone is asymmetric and crescent shaped, deflected to the left. The cell is dorso-ventrally flattened, and in ventral view, it is oval. The anterior flagellum appears in the cingulum, encircling it, while the posterior flagellum extends beyond the cell posteriorly. The nucleus is confined to the lower part of the hypocone. Respect to the chloroplast, it is considered a multi-lobed chloroplast. The measured dimensions ranged from 10.04 to 12.86 µm in length and 8.36–10.98 µm in width, that are in line with those reported for this species [2], [3].

**Fig. 2:**
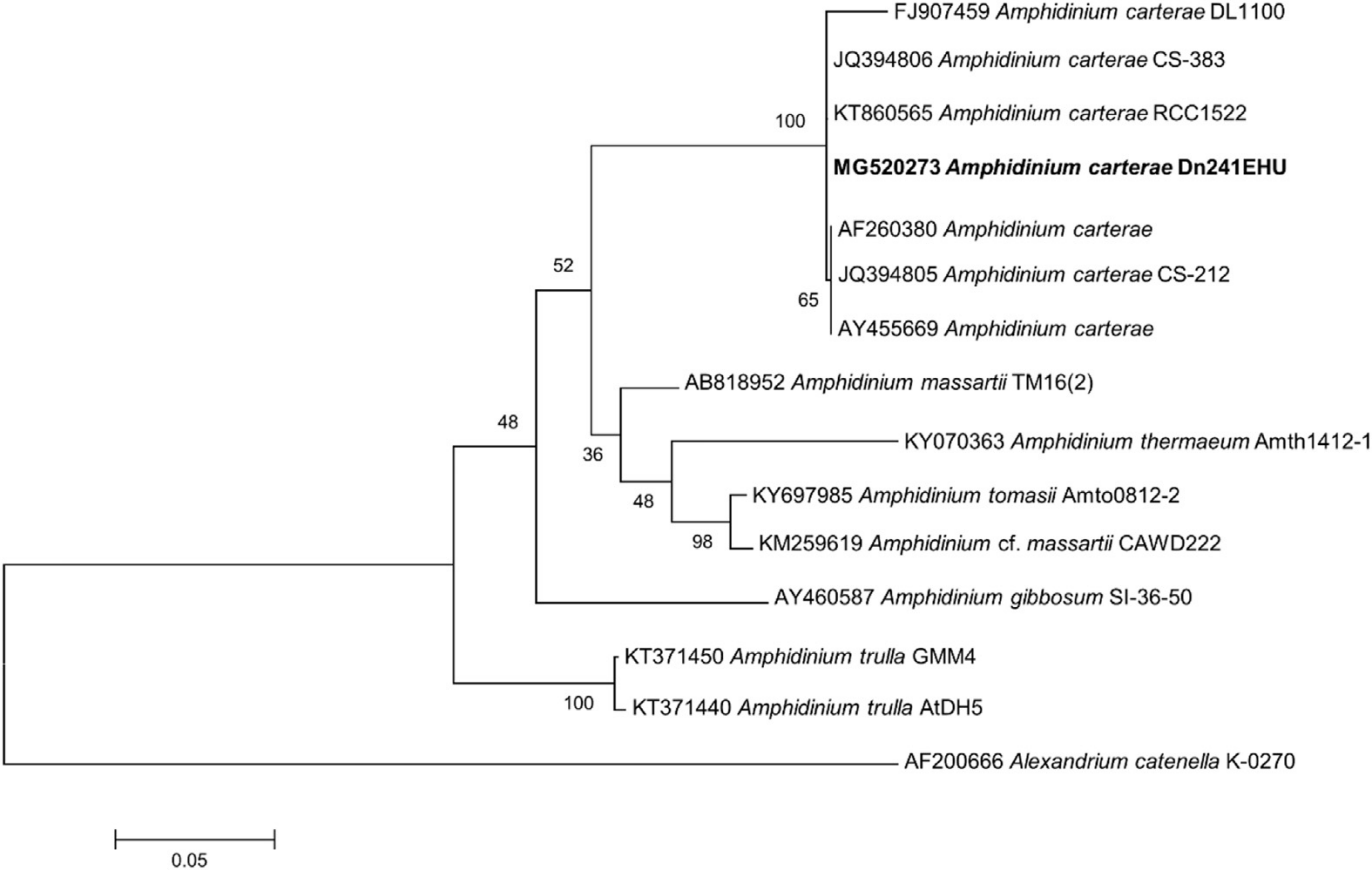
Maximum likelihood tree based on the 28S rDNA sequences of the *Amphidinium carterae* Dn241EHU (in bold) plus other relatives from GenBank. Numbers on the nodes represent ML support values (1000 replicates). *Alexandrium catenella* was used as outgroup.

The phylogenetic tree reflects a strong support of the morphological identification of the Strain Dn241EHU, which clusters in a group with other *A. carterae* strains supported by a maximum likelihood bootstrap value of 100 (Fig. 2).

## 2 Experimental design, materials, and methods

### 2.1 Isolation

The strain, used in a study for production of carotenoids and fatty acids [1], was isolated from Punta des Gas (Mallorca, Spain) (39°33′50″ N - 2°39′20″ E) in October 2013, by mouth pipetting. This technique allows isolating a unique cell to obtain clonal cultures and it is very effective with cells with no very quick movement. Cells were grown at 18 ± 2 °C under 12:12 h light–dark cycle. Illumination was provided by cold white fluorescent lamps (MASTER TL5 HO 54 W/840 SLV/40, Phillips, Holland) at the irradiance of 70 µE m^−2^ s^−1^. The strain was cultured in f/2 medium [4], with water filtrated by 0.22 µm pore size filter (GSWP, Merck, Germany) and pasteurized. The strain was maintained in glass tubes.

### 2.2 Morphological characterization

The morphological identification was carried out with two different miscroscopic techniques. Thus, cells were studied alive and photographed with a Leica DMRB (Leica, Wetzlar, Germany) light microscope, fitted with a monochromatic Sony CDD camera (Sony, Tokyo, Japan) and a NIS-Elements D ver.2.30 microscope imaging software (Nikon, Tokyo, Japan) (Fig. 1). Cells of *Amphidinium carterae* Dn241EHU were also inspected with an Andor DragonFly 200 confocal microscope (Leica, Wetzlar, Germany) (Fig. 1). Twenty cells were measured in vivo to obtain the range of the cell dimensions.

### 2.3 Molecular characterization

For molecular analyses, 20 cells were isolated by micropippeting and transferred to the PCR tube together BioMix (Bioline, London, UK) and the primers pairs D2C-D1R [5]. The loci encoding the LSU rRNA regions D1-D2 were amplified through polymerase chain reaction (PCR) with the primers pairs D2C-D1R (Scholin et al., 1994). The PCR amplification was carried out in a BIOER TC-24/H(b) thermocycler (BIOER Technology Co., Hangzhou, China) and cycling conditions were as follows: one cycle at 95 °C for 2 min, 50 °C for 30 s and 72 °C for 45 s; 35 cycles at 94 °C for 30 s, 50 °C for 90 s and 72 °C for 30 s; and a final elongation step of 72 °C for 10 min. PCR products were purified using the kit MultiScreen HTS PCR 96-well filtration system (Merck-Millipore Corp.) and quantified with the spectrophotometer Nanodrop. For sequencing reaction (forward and reverse directions), the BigDye Terminator v3.1 Cycle Sequencing Kit (Applied Biosystems, Foster City, CA) was employed, and the reading was undertaken with the automatic sequencer ABI PRISM 3130xl Genetic Analyzer. The sequences were edited using BioEdit v7.0.9 software [6].

The obtained sequence was BLASTed against the NCBI database. Phylogenetic analyses were inferred using Maximum likelihood (ML) method with MEGA6 software [7]. A total of seven sequences of *A. carterae* and seven sequences of other species of the genus *Amphidinium* were included in the phylogenetic analysis with *Alexandrium catenella* as outgroup (Fig. 2). The alignment was made with MUSCLE 3.7 [8] and the Kimura 2-parameter model was used. The 28S rDNA sequence was stored under GenBank accession number MG520273.
