# Supplementary material for: Data on the Amphidinium carterae Dn241EHU isolation and morphological and molecular characterization
Source: Data Brief. 2018 Jul 21;20:1–5. doi: 10.1016/j.dib.2018.07.036 (PMC6084018; doi:10.1016/j.dib.2018.07.036)

Dear Editor,

I confirm that the manuscript has been read and approved by all named authors and that there are no other persons who satisfied the criteria for authorship but are not listed. I further confirm that the order of authors listed in the manuscript has been approved by all of us.

I confirm that we have given due consideration to the protection of intellectual property associated with this work and that there are no impediments to publication, including the timing of publication, with respect to intellectual property. In so doing I confirm that we have followed the regulations of our institutions concerning intellectual property.

I further confirm that any aspect of the work covered in this manuscript that has involved either experimental animals or human patients has been conducted with the ethical approval of all relevant bodies and that such approvals are acknowledged within the manuscript.

Sergio Seoane

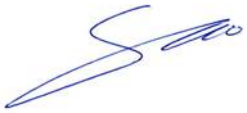

Supplement: Supplementary file 1 — Supplementary material [file mmc1.pdf]
